# Association Between Tuberculosis and Smoking

**DOI:** 10.5812/ijhrba.5215

**Published:** 2012-07-25

**Authors:** Roya Alavi-Naini, Batool Sharifi-Mood, Maliheh Metanat

**Affiliations:** 1 Infectious Diseases and Tropical Medicine Research Center, Zahedan University of Medical Sciences, Zahedan, IR Iran

**Keywords:** Tuberculosis, Smoking

## Abstract

**Background:**

The association between smoking and tuberculosis (TB), which has been proven in multiple studies with different study population ethnicity, has not yet received sufficient attention in terms of TB control.

**Objectives:**

The aim of the present study was to determine the association between TB and cigarette smoking in southeastern Iran, an endemic area for tuberculosis.

**Patients and Methods:**

This prospective case-control study conducted at a University-Affiliated Hospital (Boo-Ali Hospital, Zahedan, and Southeastern Iran) from March 2007 to March 2012 enrolled 253 TB patients and 312 healthy controls. Factors including history of cigarette smoking, duration of smoking, number of cigarettes smoked per day, consumption of other drugs (parenteral and non-parenteral), and family history of tuberculosis and smoking, were evaluated in both cases and controls. Univarate and multivariate logistic regressions were performed to compare TB cases and controls. The odds ratio (OR) and 95% confidence intervals (CI) were also estimated.

**Results:**

The results of the study revealed a significant difference between TB and control groups in relation to smoking (P < 0.0001). In multivariate logistic regression, cigarette smokers were 3.1 (95% CI: 1.4-10.3) times more frequent in TB patients compared with controls. Other factors that showed significant differences between TB patients and controls were the use of non-parenteral drugs (OR = 3.6, 95% CI: 2.2-21.4), family history of TB (OR = 6.6, 95% CI: 2.3-18.2), family history of smoking (OR = 2.8, 95% CI: 1.1-8.4), and smoking history of more than 10 years (OR = 1.6, 95% CI: 1.2-9.8).

**Conclusions:**

The present study evidenced the association between TB and smoking. It is therefore recommended to include interventions for smoking cessation in the current TB control practice.

## 1. Background

Tuberculosis (TB) is one of the leading causes of death worldwide and remains a major public health burden in many developing countries. Approximately 1.3 billion people smoke tobacco products and most of them live in low- or middle-income countries, where the burden of TB is also very high (1). The World Health Organization reported that smoking causes 9% of all deaths worldwide (2). Tobacco smoking has been shown to associate with TB infection. Moreover, cigarette smoking is also associated with negative prognosis of TB (3). Smoking may affect many organ systems, but the lungs suffer by far the most damage. Smoking damages the lungs and impacts the body’s immune system, making smokers more susceptible to TB infection. The occurrence of TB has been shown to be linked to altered immune response and multiple defects in immune cells such as macrophages, monocytes and CD4 lymphocytes (4). Other mechanisms, such as mechanical disruption of cilia function and hormonal effects, could also appear secondarily to smoking (5). Therefore, all these factors may contribute to the increased susceptibility of an individual to develop TB infection. Several studies found that significantly more current smokers developed TB and subsequently died within the follow-up period than non-smokers (6-8). It is possible that smoking increases the risk of relapse, by facilitating the persistence of M. Tuberculosis infection after treatment and augments the risk for any residual M. Tuberculosis bacilli to promote infection that would lead to disease (3). Several cross-sectional case-control studies have reported the smoking - TB association (2,9,10). The most important cohort study on this matter was conducted in India from 1991 to 2003, which presented a strong evidence of this association (11). The necessity to verify the certitude of cigarette smoking - TB association in the southeastern Iranian population constituted the rationale for the present research.

## 2. Objectives

The aim of the present study was to determine the association between TB and cigarette smoking in southeastern Iran, an endemic area for tuberculosis.

## 3. Patients and Methods

This prospective case-control study was conducted at a university-affiliated hospital (Boo-Ali Hospital, Zahedan, and southeastern Iran) from March 2007 to March 2012. Diagnosis of pulmonary TB patients was established on clinical findings, positive sputum smear for acid fast bacilli and the results of chest X-ray. Only patients who were confirmed by microbial culture were included into the study. Therefore, 253 TB patients were included in the final analysis. Three hundred and twelve normal healthy subjects who underwent the physical examination at Boo-Ali Hospital were recruited during the study period and were matched for age and sex to TB patients. The inclusion criteria for normal healthy subjects were the absence of clinical symptoms and signs suggestive for active pulmonary tuberculosis and normal chest X-ray. Factors including history of cigarette smoking, smoking duration, number of cigarettes smoked per day, consumption of other drugs (parenteral and non-parenteral), and family history of tuberculosis and smoking were registered for both TB cases and controls. Comparison between TB smokers and TB non-smokers were performed using the SPSS 17.0 software version (IBM Corp., Armonk, NY, US) and results with a P < 0.05 were considered statistically significant. Both univariate and multivariate logistic regressions were performed to compare cases and controls. The odds ratio (OR) and 95% confidence intervals (95% CI) were also estimated.

## 4. Results

Among the 253 TB patients, 123 (48.6%) were males. Ninety seven (38.3%) of TB patients were more than 50 years old and 104 (41.1%) were cigarette smokers. One hundred fifty four (49.3%) out of 312 controls were male and 116 (37.2%) of them were more than 50 years old. Cigarette smokers accounted 57 individuals (18.3%) in the control group. In univariate analysis, there was a significant difference between TB and control group, in relation to smoking (P = 0.0001). One hundred and six of the TB smokers (42%) had a history of non-parenteral consumption of drugs, especially opiate. Only 12 (4.7%) TB patients were intravenous drug users. In the control group, 51 (16.3%) were non-parenteral drug users and 10 (3.2%) used parenteral drugs. There were no statistically significant differences between the group of TB patients and controls according to use of parenteral drugs (P > 0.05), but a significant difference was shown for non-parenteral drugs (P = 0.0001). Human immunodeficiency virus (HIV) infections are not prevalent in southeastern Iran and tests for HIV detection were not requested for all patients. Therefore, only three out of 38 TB patients and one out of 24 controls, with a high risk behavior, were reported positive for HIV infection. Family history of TB and smoking were the two other risk factors that showed significant differences in TB patients and controls (P < 0.05) (*Table 1*). In multivariate logistic regression (*Table 2*), cigarette smokers were 3.1 (95% CI: 1.4-10.3) times more frequent in TB patients comparer to controls. Other factors that presented significant differences between TB patients and controls were the use of non-parenteral drugs (OR = 3.6, 95% CI: 2.2-21.4), a family history of TB (OR = 6.6, 95% CI: 2.3-18.2), a family history of smoking (OR = 2.8, 95% CI: 1.1-8.4), and history of smoking for more than 10 years (OR = 1.6; 95% CI: 1.2-9.8).

**Table 1.** General Characteristics of Tuberculosis Patients and Controls ^a ^

	TB ^b^ Patients, No. (%)	Controls, No. (%)	*P* value
Age > 50, y	97 (38.3)	116 (37.2)	0.79
Sex, Male	123 (48.6)	154 (49.3)	0.87
Cigarette smokers	104 (41.1)	57 (18.3)	0.0001
Parental drug users	12 (4.7)	10 (3.2)	0.66
Non-parenteral drug users	106 (42)	51 (16.3)	0.0001
Smoked at least 30 cigarettes per day	65 (25.7)	58 (18.6)	0.051
Smoked for at least 10 years	78 (30.8)	68 (21.8)	0.016
HIV ^b^ positive	3 (1.2)	1 (0.3)	-
Lived with anyone with TB ^b^	53 (21)	12 (3.9)	0.0001
Lived with anyone who smoke	61 (24.3)	33 (10.6)	0.0001

^a^Patients and controls numbers are presented as absolute values and percentages, *P* < 0.05 was considered statistically significant

^b^ Abbreviations: TB, tuberculosis; HIV, human immunodeficiency virus

**Table 2.** Multivariate Analysis of Common Risk Factors Related to Smoking in Tuberculosis Patients Compared to Controls ^a ^

	OR ^b^	95% CI ^b^	*P* value
Cigarette smokers	3.1	1.4 - 10.3	0.001
Smoked at least 30 cigarettes a day	1.2	1.1 - 4.8	0.09
Smoked for at least 10 years	1.6	1.2 - 9.8	0.05
Non-parenteral drug users	3.6	2.2 - 21.4	0.012
Lived with anyone with TB ^b^	6.6	2.3 - 18.2	0.001
Lived with anyone who smoke	2.8	1.1 - 8.4	0.03

^a^*P* < 0.05 was considered statistically significant.

^b^ Abbreviations: CI, confidence interval; OR, odd ratio; TB, tuberculosis

## 5. Discussion

As mentioned previously, smoking is a well-recognized major risk factor for the development of lung cancer, chronic obstructive pulmonary disease and other respiratory infections, especially TB (10). While the rates of smoking have declined in developed countries during the past few decades, they have continued to rise in less developed countries. As demonstrated in our study, cigarette smoking was three times more frequent in TB patients, compared to healthy individuals. Common risk factors related to smoking, such as smoking for more than 10 years, familial TB history, familial smoking history, and the use of non-parenteral drugs, were more frequent in TB patients, compared with controls. Smoking has been associated with TB and mortality from TB in several studies (2,6,7,12). A 14-year prospective cohort study (1992–2006) carried out in South Korea from 1992 to 2006 revealed evidences that smoking increases the incidence of TB, the mortality rates from this disease, and TB recurrence (7). The adjusted risk of TB deaths among bidi smokers was 2.60 (95% CI: 2.02 - 3.33) times higher than for never-smokers, in a prospective study in Mumbai, India, with a 4 years duration from 1999 to 2003. In this study, approximately 32% of TB deaths could be attributable to bidi smoking (11). In another study carried out in southern India, smoking, which increases the incidence of clinical TB, was the cause of half of the male TB deaths, and of a quarter of all male deaths in middle age (6). Smoking (OR = 2.53, 95% CI: 1.23–5.21) and living in an area where the family health program was not implemented (OR = 3.61, 95% CI: 1.46–8.93) were found to be independently associated with the relapse of TB, as indicated by the report of Batista et al. in the northeast region of Brazil (13). Similarly to our study, other researches carried out in the United Kingdom, Taiwan and China, revealed the strong association between TB and smoking, and the reduction of TB risks by smoking cessation (9,12,14-16). Smoking has been shown to significantly associate with a delay in sputum smear conversion time. In addition, exposure to second-hand smoke can also increase the risk for both TB infection and development of active TB disease, among children and adults. Therefore, TB patients who smoke at home are also placing their families at a greater risk of TB infection. Similar to other researches, the association between TB and smoking was demonstrated in our study. It is therefore recommended to include interventions for smoking cessation in the current TB control practice.
